# Hypothalamic Kisspeptin Neurons Regulates Energy Metabolism and Reproduction Under Chronic Stress

**DOI:** 10.3389/fendo.2022.844397

**Published:** 2022-05-24

**Authors:** Yinqiong Huang, Qinyu Liu, Guifeng Huang, Junping Wen, Gang Chen

**Affiliations:** ^1^Department of Endocrinology, Shengli Clinical Medical College of Fujian Medical University, Fuzhou, China; ^2^Department of Endocrinology, The Second Affiliated Hospital of Fujian Medical University, Quanzhou, China; ^3^Department of Endocrinology, Fujian Provincial Hospital, Fuzhou, China; ^4^Fujian Provincial Key Laboratory of Medical Analysis, Fujian Academy of Medical, Fuzhou, China

**Keywords:** reproductive function, energy metabolism, glucocorticoid, restraint stress, Kisspeptin

## Abstract

**Background:**

Stress activates the hypothalamic-pituitary-adrenal (HPA) axis, affecting energy homeostasis and reproduction. The aim of this study was to investigate whether stress affected energy metabolism and reproduction through the glucocorticoid receptor on Kisspeptin neurons in the hypothalamus.

**Methods:**

Four groups included control group, chronic restraint stress group, Kisspeptin specific glucocorticoid receptor knock out group (KGRKO) and KGRKO+stress group. Body weight, food intake, estrous cycle of female mice, serum sex hormone levels, serum corticosterone and prolactin, Kisspeptin expression in the hypothalamus were measured.

**Results:**

The restraint stress group showed a significant weight loss compared with the control group. KGRKO+restraint stress group had a reduced weight loss, suggesting that restraint stress might partially affect the energy metabolism through GR on Kisspeptin neurons. In terms of reproductive function, the restraint stress group and the KGRKO+restraint stress group showed missing pre-estrus period or prolonged estrous cycles. Serum LH and FSH in KGRKO + restraint stress group decreased significantly compared with KGRKO group. However, no significant difference in the level of serum testosterone was observed. After restraint stress, the levels of serum cortisol and prolactin in male and female mice were significantly higher than the control group, and the hypothalamus Kiss1 gene mRNA expression and Kisspeptin protein expression were significantly decreased.

**Conclusion:**

Chronic restraint stress induced weight loss and negative changes in reproduction, which were partially mediated by glucocorticoid receptor on Kisspeptin neurons in the hypothalamus.

## Introduction

With the rapid development of modern society, people are under great pressures. Stress may lead to physiological responses including decreased appetite, increased blood pressure and heart rate, reproductive dysfunction, insomnia, and even anxiety and depression, which brings huge economic burdens and losses to society.

As a stress response, the hypothalamic-pituitary-adrenal axis (HPA) is activated and glucocorticoids (glucocorticoid, GC) are secreted through the adrenal glands (1). long-term improper stress can affect normal physiological functions including energy metabolism and reproductive function. Studies have found that long-term exposure to stress reduces the body weight and food intake of rodents. Rats exposed to acute or chronic restraint stress have significant changes in blood lipids and lipoprotein levels, and plasma free fatty acids and cholesterol levels increase. And the triglyceride level is reduced. Disorders of reproductive function have also been observed in patients with anorexia nervosa (2) and girls with severe psychological trauma (3). The common feature of these people is that they have been activated on the HPA axis for a long time, and their cortisol levels are higher than normal. In population studies, it was also found that the blood cortisol level of prepubertal girls was positively correlated with the age of menarche (2). Famine exposure is also a kind of stress response. Our research team analyzed the relationship between famine exposure and reproductive function in 2868 women in a previous study. We found that exposure to famine during childhood increases the incidence of premature menopause and even premature ovarian failure (4). In animal experiments, it can also be observed that the mother is exposed to glucocorticoids during pregnancy and the estrus cycle is delayed after the offspring is born (5). Therefore, stress will affect the body’s energy metabolism and reproductive function.

What are the specific mechanisms by which stress affects energy metabolism and reproductive function? Studies showed that central mechanism, especially the hypothalamus, might be involved (6).

Kisspeptin neurons are mainly distributed in the hypothalamic arcuate nucleus (ARC), and the third paraventricular anterior ventral nucleus (AVPV) (7). It can directly act on hypothalamic GnRH neurons to promote the secretion of GnRH, resulting in hypothalamic-pituitary-gonadal (HPG) Activate, causing reproductive function to be affected. Kisspeptin plays an important role in reproductive function (8). One of the reasons for hypogonadotropic sexual dysfunction is the mutation of Kiss1 gene. Therefore, Kisspeptin is the key to start and maintain the function of the HPG axis (9). In addition, there are insulin and adiponectin receptors on Kisspeptin neurons, which can sense metabolic changes and regulate energy (10). Therefore, Kisspeptin may be a bridge between energy metabolism and reproductive function.

Glucocorticoid receptor (GR) is expressed on Kisspeptin neurons in the mouse hypothalamus (11), which suggests that the regulation of glucocorticoids on reproductive function may be through the Kisspeptin neurons in the hypothalamus. Kinsey-Jones et al. (12) observed that the expression of kisspeptin mRNA in the hypothalamus of female rats decreased under different stress conditions, and the results of our group’s previous studies also confirmed that dexamethasone can inhibit hypothalamic GT1-7 neuronal cells (GnRH, Kisspeptin Both expression and secretion) The transcriptional expression of Kiss1 gene mRNA and the expression level of Kisspeptin protein, and the glucocorticoid receptor blocker RU486 can antagonize this effect. These findings suggest that hypothalamic Kisspeptin neurons may be stress-affected energy A new important central target of metabolism and reproductive function.

In this study, we established a mouse model of chronic restraint stress to observe the influence of chronic restraint stress (CRS) on energy metabolism and reproductive function, as well as the influence of kisspeptin expression in the hypothalamus. We further constructed the Kisspeptin neuron-specific glucocorticoid receptor knockout mice (Kisspeptin specific glucocorticoid receptor knock out, KGRKO) undergo CRS which helps to reveal the role of stress in regulating energy metabolism and reproductive function through the central nervous system, to further elucidate the interaction mechanism between HPA and HPG axis and the relationship between stress-induced hypercortisolemia and reproductive diseases.

## Materials and Methods

### Laboratory Animals and Reagents

Kisspeptin specific glucocorticoid receptor knock out (KGRKO) mice was accomplished by crossing mice engineered with lox P sites flanking exons 1C and 2 of the mouse GR gene (GRloxP) with mice expressing Cre recombinase driven by the Kiss1 Cre promoter (Kiss1Cre) on a C57BL/6J background. Male and female KGRKO mice (homozygous for GR flox [GR flox/flox] and expressing Kiss1Cre), here denoted as GRflox/floxKIss1cre, and littermate control mice (GRflox/floxKiss1cre) containing only the GR flox allele (no Kiss1-Cre transgene) were used for all studies and were 8–10 weeks old at the beginning of each experiment. To generate litters expressing both of these genotypes, the female breeders were GRflox/floxKiss1cre-, whereas the male breeders were GRflox/floxKiss1cre+.

All mice were housed under standard conditions (constant temperature, constant humidity conditions, and a 12-h light/dark cycle), 5 mice a cage, with free access to food and water. The study followed the National Guidelines for Laboratory Animal Welfare and was approved by the Experimental Animal Ethics Committee of Fujian Medical University.

### Establishment of Mice Model

There were four groups, that were control group, chronic restraint stress group (stress group), Kisspeptin specific glucocorticoid receptor knock out group (KGRKO group) and KGRKO+stress group, 10 mice for each group. Twenty mice of wildtype mice and KGRKO mice were randomly divided into control and stress group, respectively.

Stress mice were placed in a 50ml centrifuge tube. The top and side walls of the centrifuge tube have small holes with a diameter of 0.5 cm to ensure air flow. The restraint stress is performed for 1 hour from 9:00 to 10:00 in the morning. During the restraint period, the animal has no food or water. After 1 hour restraint, the mice were immediately returned to their cages, and they were free to eat and drink. The unrestrained stressed mice (control group) were kept in the cage.

Investigators could not be blinded to the mouse strain due to that the stress group mice were placed in a tube.

### Serum Measurement

Serum sex hormone levels, serum corticosterone and prolactin were measured with ELISA Kit, following the manufactory instructions.

### Expression of Kisspeptin in the Hypothalamus by Immunofluorescence

The animals were perfused with phosphate buffered solution (PBS) at a pH of 7.4 by a cannula into the left ventricle after anaesthesia, followed by 4% paraformaldehyde. After perfusion, the brains were immediately removed and were fixed in 4% paraformaldehyde in PBS at 4°C for 12 h and passed through 20 and 30% sucrose gradients prior to embedding in optimum cutting temperature compound (OCT). 20μm tissue sections were air-dried at −20°C and moved to −80°C for long-term storage. Immunofluorescence was performed according to the manufacturer’s instructions for fixed-frozen tissue. Glass coverslips were fixed with 4% paraformaldehyde, 0.1% triton X-100 permeabilized, blocked with 1% BSA, and incubated with the Kisspeptin antibody (Mouse monoclonal antibody; Abcam, USA; 1: 500) at 4°C overnight. After washes with 1×PBS, cells were incubated with the corresponding secondary antibody conjugated with Alexa Fluor^®^ 647 (Donkey polyclonal Secondary Antibody to mice IgG - H&L; Abcam, USA; 1:100) in dark for 1 h and analyzed using Inverted fluorescence microscope (Leica Microsystems Ltd. CH-9435 Heerbrugg. Type: DFC425 C(12730222). Serial No: 533481211. Leica, Germany). 4′, 6-diamidino-2-phenylindole (DAPI) was used to label nucleus. ImageJ was used to calculate the expression of Kisspeptin fluorescence intensity per scaffold. At least 3 slides were examined in each treatment group for each experiment. A comparison of the fluorescence intensity of Kisspeptin between the indicated groups was performed.

### Quantitative RT-PCR (qRT-PCR)

At the end of each experiment, a microdissection procedure was used to isolate hippocampus. Total RNA was extracted with TRIzol (RNAiso Plus)method (Takara, Japan). RNA was reversed transcribed into cDNA using the two-step method with PrimeScript™ RT reagent Kit with gDNA Eraser (Takara, Japan), according to the manufacturer’s instructions. mRNA qRT-PCR was performed with the TB Green™ Premix Ex Taq™ (TliRNaseH Plus) (Takara, Japan) according to the manufacturer’s instruction. The procedure was 95°C for 1 min; 95°C for 15 s and 60°C for 34 s, for 40 cycles; 95°C for 15 s, 60°C for 1 min and 95°C for 15 s. The primers were shown in **Table 1**.

**Table 1:** Primers of qRT-PCR.

Gene		Primers sequence
β-actin	Forward	5’ CTACCTCATGAAGATCCTGACC 3’
	Reverse	5’ CACAGCTTCTCTTTGATGTCAC 3’
GR^flox/flox^	Forward	5’-ATGCCTGCTAGGCAAATGAT-3’
	Reverse	5’-TTCCAGGGCTATAGGAAGCA-3’
Kisspeptin	Forward	AGCTGCTGCTTCTCCTCTGT-3’
	Reverse	AGGCTTGCTCTCTGCATACC-3’

β-actin was used as mRNA reference gene, with the 2−ΔΔCt method used for quantitation. Triplicate experiments were performed and repeated at least 3 times.

### Statistical Analyses

All statistical analyses were performed using the SPSS Statistics 20 software. Data have been expressed in terms of mean ± standard deviation. Statistical significances between two groups of data were determined using unpaired, two-tailed Student’s *t*-test. A *P* value >0.05 was not considered significant, *P* value <0.05 was labeled as (*), *P* value <0.01 was labeled as (**), *P* value <0.001 was labeled as (***).

## Results

### The Effect of Chronic Restraint Stress on the Body Weight of KGRKO Mice

In order to understand the effect of GR signals on Kisspeptin neurons on the body weight of mice under chronic restraint stress, we performed chronic restraint stress on mice for 28 days and monitored their body weight every day.

**Figure 1:**
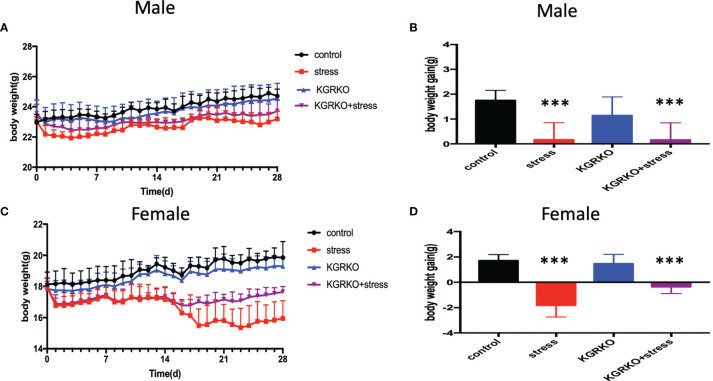
The effect of chronic restraint stress on the body weight of KGRKO mice. control: control group, stress: restraint stress group. KGRKO group: Kisspeptin neuron-specific GR knockout mice. KGRKO+stress group: Kisspeptin neuron-specific GR knockout mice restraint stress group. **(A, B)** The effect of chronic restraint stress on the body weight of male KGRKO mice. **(C, D)** The effect of chronic restraint stress on the body weight of male KGRKO mice. ***Represents the comparison with the control group, P<0.001.

The mice were divided into four groups: control group, restraint stress group, KGRKO group, KGRKO+ restraint stress group, and weight and food intake were measured every day for 28 days. As shown in **Figures 1A, B**, after 28 days of intervention, the restraint stress group compared with the control group, and the KGRKO+ restraint stress group compared with the KGRKO group, the body weight was significantly reduced. Compared with the restraint stress group, the weight loss of the KGRKO+ restraint stress group was reduced.

As shown in **Figures 1C, D**, after 28 days of intervention, the restraint stress group compared with the control group, and the KGRKO+ restraint stress group compared with the KGRKO group, the body weight was significantly reduced. Compared with the restraint stress group, the weight loss of the KGRKO+ restraint stress group was reduced, suggesting that the influence of restraint stress on energy metabolism may partly act through the GR on the Kisspeptin neurons.

### The Effect of Chronic Restraint Stress on the Estrous Cycle

In order to understand the influence of GR signals on Kisspeptin neurons on the estrous cycle of mice under chronic restraint stress, we performed vaginal smears every day to observe the estrous cycle 10 days before restraint stress.

**Figure 2:**
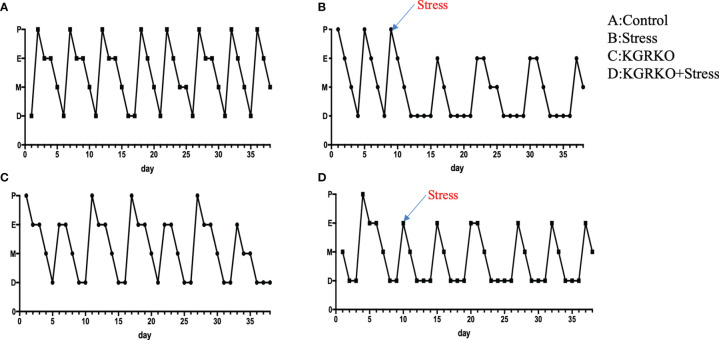
The effect of chronic restraint stress on the estrous cycle of female KGRKO mice. Estrous cycle included 4 states, P, Proestrus; E, Estrus; M, Metestrus; D, Diestrus. **(A)** control group, **(B)** restraint stress group. **(C)** KGRKO group, Kisspeptin neuron-specific GR knockout mice group. **(D)** KGRKO+ restraint stress group, Kisspeptin neuron-specific GR knockout mice restraint stress group.

From 10 days before restraint stress to 28 days before restraint stress ended, lasting 38 days, vaginal smears were performed every day. As shown in **Figure 2**, the control group showed a regular estrus cycle of 4–5 days, while the restraint stress group and KGRKO+ restraint stress group showed a lack of proestrus or a prolonged estrus cycle.

### The Effect of Chronic Restraint Stress on Serum Sex Hormone

**Figure 3:**
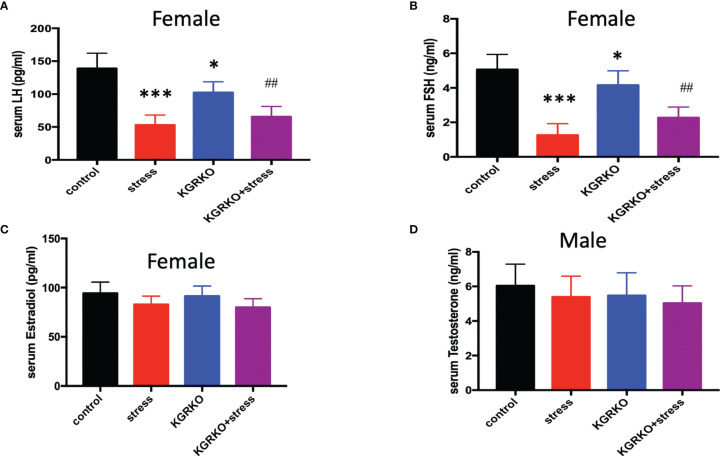
The effect of chronic restraint stress on sex hormones in KGRKO mice. **(A)** LH level in female mice; **(B)** FSH level in female mice; **(C)** Estrodiol level in female mice; **(D)** Testosterone level in male mice; control: control group, stress: restraint stress group. KGRKO group: Kisspeptin neuron-specific GR knockout mice. KGRKO+stress group: Kisspeptin neuron-specific GR knockout mice restraint stress group. *Represents the comparison with the control group, P<0.05. ^##^Represents the comparison with the KGRKO group, P < 0.01. ***Represents the comparison with the control group, P < 0.001.

In order to understand the influence of GR signals on Kisspeptin neurons on the levels of sex hormones in mice under chronic restraint stress, we collected blood from the orbit after the experiment, and separated serum to detect LH, FSH and estrogen levels after centrifugation. As shown in **Figures 3A–C**, compared with the control group, the female mice restraint stress group and the KGRKO group showed a significant decrease in serum LH and FSH, and the difference was statistically significant, while the serum estrogen level had no significant difference (P> 0.05). Compared with the KGRKO group, the serum LH and FSH of the KGRKO+ restraint stress group decreased, and the difference was statistically significant (P<0.05), but there was no significant difference in the serum estrogen level (P>0.05). As shown in **Figure 3D**, there was no significant difference in serum testosterone levels between male restraint stress group, KGRKO group and KGRKO+ restraint stress group (P>0.05).

### Effect of Chronic Restraint Stress on Serum Cortisol Level in Mice

**Figure 4:**
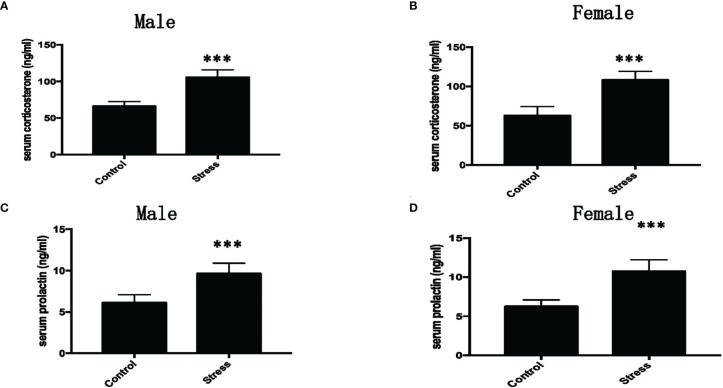
The effect of chronic restraint stress on serum cortisol and prolactin. **(A)** Cortisol in male mice; **(B)** Cortisol in female mice; **(C)** Prolactin in male mice; **(D)** Prolactin in female mice. control: control group, stress: restraint stress group. n=10. ***Represents the comparison with the control group, P<0.001.

In order to explore the effects of chronic restraint stress on cortisol and prolactin in mice, after 28 days of restraint stress, the mice were subjected to orbital blood collection, and the serum was separated to detect the levels of cortisol and prolactin after centrifugation, as shown in **Figures 4A, B**. It can be seen that the serum cortisol in the restraint stress group was significantly higher than that in the control group (P<0.001). As shown in **Figures 4C, D**, we can see that whether it is male or female, the serum prolactin of the restraint stress group was significantly higher than that of the control group (P<0.001).

### Effect of Chronic Restraint Stress on the Expression of Kisspeptin Protein in Mouse Hypothalamus

**Figure 5:**
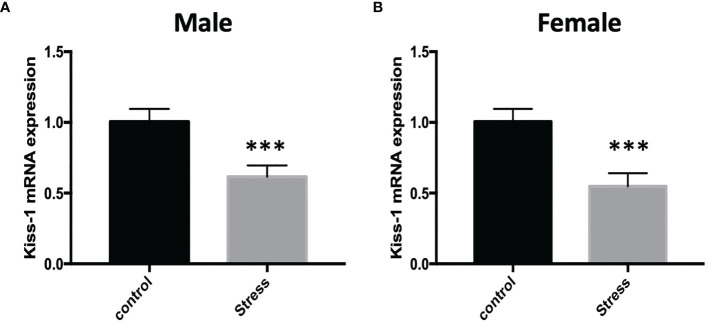
The effect of chronic restraint stress on the expression of Kiss1 mRNA in the hypothalamus. control: control group, stress: restraint stress group. n=5. ***Represents the comparison with the control group, P<0.001. **(A)** Kiss-1 mRNA expression after chronic restraint stress in male mice; **(B)** Kiss-1 mRNA expression after chronic restraint stress in female mice.

**Figure 6:**
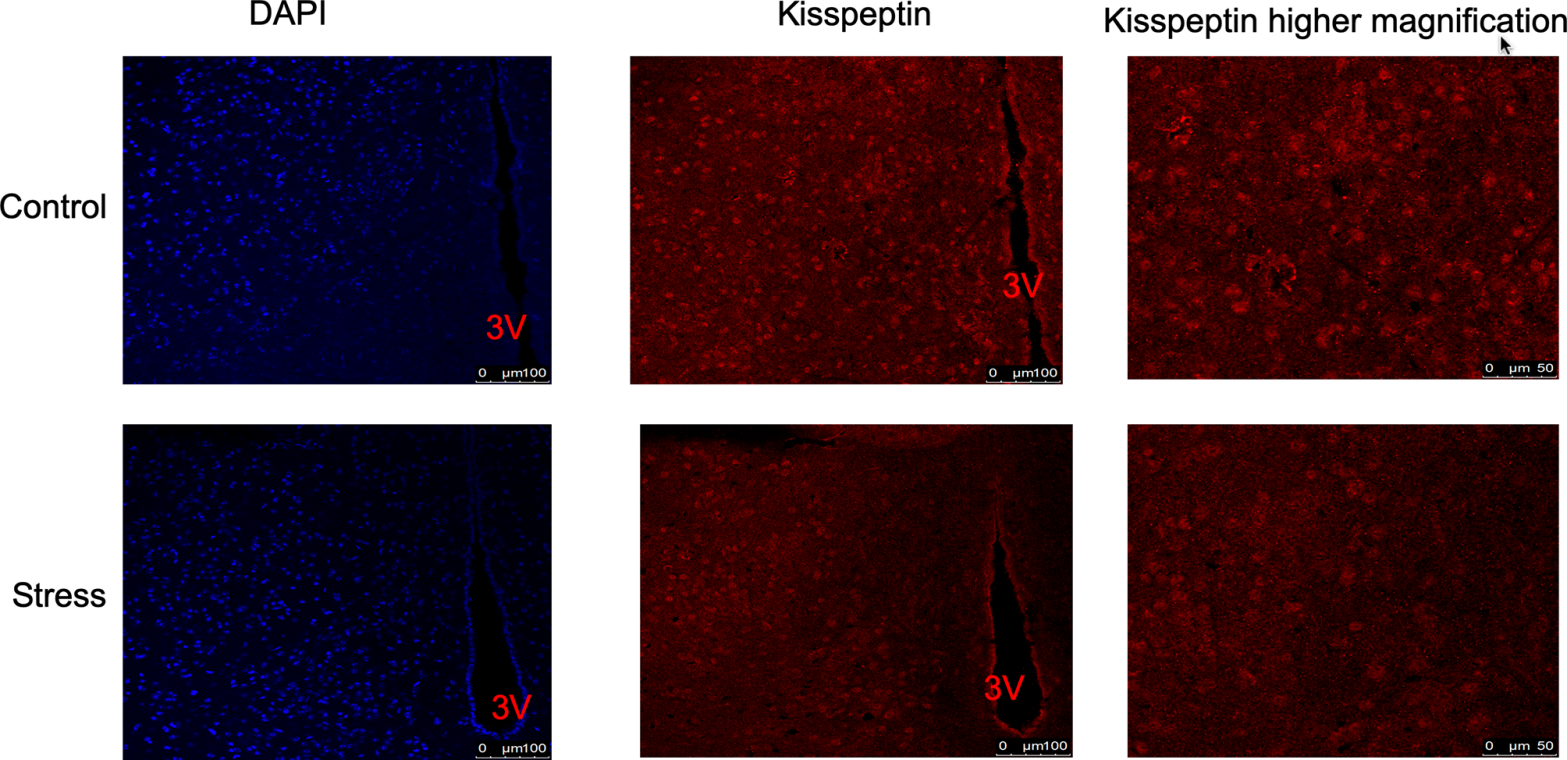
The effect of chronic restraint stress on the expression of Kisspeptin protein in the hypothalamus. The expression of Kisspeptin was detected by immunofluorescence. Blue represents DAPI nuclear staining, and red represents Kisspeptin immunofluorescence staining. control: control group, stress: restraint stress group. 3V, Third ventricle. n=3.

In order to explore whether chronic restraint stress affects the expression of KiSS1 gene mRNA in hypothalamus, at the end of the experiment, we isolated mouse hypothalamus, extracted hypothalamic mRNA, and detected the expression of KiSS1 gene mRNA in hypothalamus by Real-time PCR. The results showed that compared with the control group, whether it was male or female mice, after 28 days of restraint stress, the expression of KiSS1 gene mRNA in the hypothalamus decreased significantly, as shown in **Figure 5**. And the expression of Kisspeptin protein in hypothalamus was detected by immunofluorescence method. As shown in **Figure 6**, the expression of Kisspeptin in the hypothalamus of the chronic restraint stress group was lower than that of the control group, which is consistent with the effect of chronic restraint stress on the transcription and expression of hypothalamic Kiss1mRNA.

## Discussion

The main findings in our present study include (1) Chronic restraint stress induced weight loss in mice and reversed body weight gain induced by high-fat diet. Chronic restraint stress played a negative role in regulating reproductive function. (2) the KGRKO+restraint stress group had a reduced weight loss compared with the restraint stress group,; (3) the restraint stress group and the KGRKO+restraint stress group showed missing pre-estrus period or prolonged estrous cycles. (4) Serum LH and FSH in KGRKO + restraint stress group decreased significantly compared with KGRKO group.

Stress activates the hypothalamic-pituitary-adrenal axis, promotes the synthesis and release of glucocorticoids, thereby affecting the expression and regulation of target tissue genes. Mice under chronic stress showed elevated basal cortisol levels (13, 14), and this result may reflect the changes in the body’s sensitivity to the negative feedback effects of circulating glucocorticoids (15). In this study, we found that the levels of cortisol and prolactin in restraint stress mice increased, which is consistent with the physiological response that repeated stress caused the HPA axis to be activated (16–18).

The HPA axis is involved in the regulation of energy metabolism. Under stress, the synthesis and release of adrenal glucocorticoids increase. Food intake and many metabolic processes are mediated by glucocorticoids. Therefore, adrenal glucocorticoids mediate changes in the body’s energy and metabolic requirements (19). For example, glucocorticoids can promote liver gluconeogenesis to ensure energy supply. In addition to stress affecting energy metabolism (20), it also has an inhibitory effect on the hypothalamic-pituitary-gonad (HPG) axis. We already know that glucocorticoids can act on the center and play a negative feedback effect on the HPA axis. So what is the mechanism in the center? Studies have found that the Kisspeptin neurons in the ARC region of the hypothalamus may be a bridge between the HPA axis and the energy metabolism and HPG axis (21, 22).

Kisspeptin is expressed in the placenta, hypothalamus, pituitary gland and gonads (23, 24), and previous studies have confirmed its role in reproductive function. KiSS1/KiSS1R gene inactivation mutations can lead to idiopathic hypogonadotropic hypogonadism (IHH) (25). Animal experiments also confirmed the expression of Kiss1R in GnRH neurons of mice (26). GnRH antagonists were injected into the lateral ventricle of adult mice, and it was observed that the effect of Kisspeptin in promoting the release of LH was inhibited. While the expression of Kiss1 in the hypothalamus is reduced, a disordered estrus cycle also appears (27). The above studies have shown that Kisspeptin plays an important role in the physiological function of GnRH, pubertal development and reproductive function, and is an important factor in maintaining the HPG axis (9).

In addition to its role in the reproductive system (28), since Kisspeptin neurons are expressed in the arcuate nucleus, attention is also paid on its role in energy metabolism in recent years. There are leptin receptors on Kisspeptin neurons. It was found that in low leptin animal models the expression of Kisspeptin decreased. In case of fasting, GnRH release can be reduced, leading to hypogonadotropic hypogonadism, and a decrease in the expression of Kiss1 gene and Kisspeptin protein, and exogenous supplementation of Kisspeptin can improve fasting low levels of gonadotropins. In addition, Kisspeptin is expressed in many peripheral tissues (including pituitary, pancreas, and adipose tissue) related to energy balance and reproduction (29, 30). Kisspeptin is also believed to affect the secretion of metabolic hormones, including aldosterone, adiponectin, insulin, growth hormone, oxytocin, and prolactin (31, 32). Hypothalamic AMPK signaling plays a key role in the metabolic control of puberty, acting *via* a repressive modulation of ARC Kiss1 neurons in conditions of negative energy balance (33). All these observations suggest that Kisspeptin has a potential connection between metabolic state and reproductive function.

Recently, some scholars reported that the use of continuous light stimulation or day and night intervention in rats, immunohistochemistry found that the expression of kisspeptin in the hypothalamus decreased (34), suggesting that the expression of kisspeptin in the hypothalamus will be inhibited under stress. The reproductive dysfunction caused by stress may be related to the inhibition of Kisspeptin neurons in ARC (35, 36).

In addition, glucocorticoid receptor (GR) is expressed on the mouse hypothalamic Kisspeptin neuron cells (11), while the expression of Kisspeptin mRNA in the hypothalamus of female mice is reduced under different stress conditions (31), and our previous studies have also confirmed that dexamethasone can inhibit the transcriptional expression of Kiss1 mRNA and the expression level of Kisspeptin protein in hypothalamic GT1-7 neuronal cells (GnRH and Kisspeptin are both expressed and secreted), and the glucocorticoid receptor blocker RU486 can antagonize this effect. These findings suggest that kisspeptin neurons in the hypothalamus may be a new and important central target that stress affects energy metabolism and reproductive function.

We confirmed in this study that restraint stress can lead to changes in food intake and weight, and lead to disorders of reproductive function, while restraint stress increases cortisol levels. However, it is not clear whether Kisspeptin is involved in the central effect of stress. Therefore, in this study, we used the Cre-loxp system to construct Kiss1 neuron-specific GR knockout mice to further explore whether restraint stress affects energy metabolism and reproductive function, and whether GR plays a role in Kisspeptin neurons.

In this study, we first constructed Kisspeptin neuron-specific GR knockout mice, and tested the role of GR signals on Kisspeptin neurons in the mouse HPA axis in the rhythm of mouse serum cortisol. We collected morning and afternoon blood samples to assess the rhythm of cortisol levels. We found that compared with control GRflox/flox/Kiss1Cre mice, the trough and peak values ​​of serum cortisol in female GRflox/flox/Kiss1Cre+ mice were not significantly different, suggesting that the GR signaling pathway on Kisspeptin neurons is more effective than normal HPA in male mice. The axis may not be a critical signal. In female KGRKO mice, they showed different HPA axis phenotypes. Compared with the control GRflox/flox/Kiss1Cre-mice, female GRflox/flox/Kiss1Cre++ mice had a significantly higher morning serum cortisol trough, but no significant difference in the afternoon serum cortisol peak.

In addition, we also compared the changes of stress on the body weight of KGRKO mice. We found that the body weight of KGRKO restraint stress mice was significantly lower than that of KGRKO mice. Compared with the restraint stress group, the weight loss of the KGRKO+ restraint stress group was reduced. Although KGRKO mice still showed weight loss after restraint stress, compared with control mice, the degree of base body weight loss was less than that of control mice after restraint stress. It is suggested that GR on Kisspeptin neurons is not a key part in the change of energy metabolism caused by restraint stress, but the influence of restraint stress on energy metabolism may partly act through GR on Kisspeptin neurons. In terms of reproductive function, the restraint stress group and KGRKO+ restraint stress group showed loss of preestrus or prolonged estrus cycle. The serum LH and FSH of the KGRKO+ restraint stress group decreased compared with the KGRKO group, and the difference was statistically significant. The above results suggest that restraint stress may partially affect energy metabolism through the GR signaling pathway on Kisspeptin neurons, and its specific mechanism needs to be confirmed by further studies.

In conclusion, this study confirmed that chronic restraint stress has a negative effect on the body’s energy metabolism and reproductive function, which is reflected in the weight gain and decrease of mice after chronic restraint stress, as well as reproductive dysfunction. Our further study confirmed that chronic restraint stress caused an increase in cortisol levels and a down-regulation of Kiss1 gene transcription in the hypothalamus. Finally, by constructing Kisspeptin neuron-specific GR knockout mice, we confirmed that chronic restraint stress may partially affect energy metabolism through the GR signaling pathway on Kisspeptin neurons. However, how chronic restraint stress affects peripheral energy metabolism and reproductive function through hypothalamic kisspeptin. In addition to kisspeptin, whether there are other central mechanisms involved, these issues need to be further studied.

## Data Availability Statement

The raw data supporting the conclusions of this article will be made available by the authors, without undue reservation.

## Ethics Statement

The animal study was reviewed and approved by the Experimental Animal Ethics Committee of Fujian Medical University.

## Author Contributions

YH, JW, and GC conceptualized and designed these studies, performed them, and wrote the manuscript. YH contributed through data analyses, data interpretation, and manuscript preparation. All authors contributed to manuscript revision and read and approved the submitted version.

## Funding

Funding for this work was supported by the Chinese National Natural Science Foundation (No. 81570706, 82070878, 81970680).

## Conflict of Interest

The authors declare that the research was conducted in the absence of any commercial or financial relationships that could be construed as a potential conflict of interest.

## Publisher’s Note

All claims expressed in this article are solely those of the authors and do not necessarily represent those of their affiliated organizations, or those of the publisher, the editors and the reviewers. Any product that may be evaluated in this article, or claim that may be made by its manufacturer, is not guaranteed or endorsed by the publisher.
